# Therapy-resistant and -sensitive lncRNAs, SNHG1 and UBL7-AS1 promote glioblastoma cell proliferation

**DOI:** 10.1155/2022/2623599

**Published:** 2022-03-11

**Authors:** Mei Cao, Rong Ma, Huaqing Li, Juan Cui, Chi Zhang, Jian Zhao

**Affiliations:** ^1^Core Laboratory, School of Medicine, Sichuan Provincial People's Hospital Affiliated to University of Electronic Science and Technology of China, Chengdu 610072, China; ^2^Department of Pharmacology, School of Basic Medicine, Tongji Medical College, Huazhong University of Science and Technology, Wuhan 430030, China; ^3^Department of Computer Science and Engineering, University of Nebraska-Lincoln, Lincoln, NE 68588, USA; ^4^Department of Radiation Oncology, Fred & Pamela Buffet Cancer Center, University of Nebraska Medical Center, Omaha, Nebraska, USA; ^5^Key Laboratory of Biological Resource and Ecological Environment of Chinese Education Ministry, College of Life Sciences, Sichuan University, No. 24 South Section 1, Yihuan Road, Chengdu 610064, China

## Abstract

The current treatment options for glioblastoma (GBM) can result in median survival of 15-16 months only, suggesting the existence of therapy-resistant factors. Emerging evidence suggests that long non-coding RNAs (lncRNAs) play an essential role in the development of various brain tumors, including GBM. This study aimed to identify therapy-resistant and therapy-sensitive GBM associated lncRNAs and their role in GBM. We conducted a genome-wide transcriptional survey to explore the lncRNA landscape in 195 GBM brain tissues. Cell proliferation was evaluated by CyQuant assay and Ki67 immunostaining. Expression of MAD2L1 and CCNB2 was analyzed by western blotting. We identified 51 lncRNAs aberrantly expressed in GBM specimens compared with either normal brain samples or epilepsy non-tumor brain samples. Among them, 27 lncRNAs were identified as therapy-resistant lncRNAs that remained dysregulated after both radiotherapy and chemoradiotherapy; while 21 lncRNAs were identified as therapy-sensitive lncRNAs whose expressions were reversed by both radiotherapy and chemoradiotherapy. We further investigated the potential functions of the therapy-resistant and therapy-sensitive lncRNAs and demonstrated their relevance to cell proliferation. We also found that the expressions of several lncRNAs, including SNHG1 and UBL7-AS1, were positively correlated with cell-cycle genes' expressions. Finally, we experimentally confirmed the function of a therapy-resistant lncRNA, SNHG1, and a therapy-sensitive lncRNA, UBL7-AS1, in promoting cell proliferation in GBM U138MG cells. Our *in vitro* results demonstrated that knockdown of SNHG1 and UBL7-AS1 showed an additive effect in reducing cell proliferation in U138MG cells.

## 1. Introduction

Glioblastoma (GBM) is the most aggressive type of brain cancer [1]. The current treatment options, such as surgery, radiation, and chemotherapy in combination, can result in median survival of 15-16 months only [2]. There is thus an urgent need to identify novel therapeutic targets for the treatment of GBM. Long non-coding RNAs (lncRNAs) have emerged as critical players in the pathogenesis and development of various cancers, including malignant brain tumors such as GBM [3]. Studies have demonstrated that lncRNAs CRNDE [4], H19 [5], NEAT1 [5], LINC00461 [6], and HOTAIR [7] play an essential role in regulating both cell proliferation and migration in GBM. Interestingly, loss-of-function studies have demonstrated that inhibition of lncRNAs MIR22HG [8], SPRY4-IT1 (H. [9]), XIST [10], CCAT2 [11], LUCAT1 [12], and AB073614 (J. [13]) successfully reduced certain GBM features, including proliferation and migration. Additionally, knockdown of H19 has been shown to sensitize human glioma cells to temozolomide therapy [14]. These findings suggested that lncRNAs could be effective targets for the treatment of GBM.

While the use of GBM cell lines and animal models in some of the studies could limit the clinical significance, there are existing gene expression datasets on human cancer biopsies that could provide vital information on the expression of lncRNAs in GBM patients. Several gene expression profiles on GBM specimens of patients have been generated using Affymetrix Human Genome U133 Plus 2.0 Array, which was designed mainly to detect mRNAs. Reannotation analysis suggests that this Affymetrix microarray can also detect 3053 lncRNAs [15].

The current study was aimed at exploring the lncRNA landscape in GBM patients. We identified 51 lncRNAs aberrantly expressed in GBM specimens compared with either normal brain samples or epilepsy non-tumor brain samples. Among them, the expressions of 27 lncRNAs were resistant to both radiotherapy and chemoradiotherapy; while the expressions of 21 lncRNAs were reversed by both radiotherapy and chemoradiotherapy. We further investigated the potential functions of the therapy-resistant and therapy-sensitive dysregulated lncRNAs and demonstrated their relevance to cell proliferation. We also found that the expressions of several lncRNAs, such as SNHG1 and UBL7-AS1, were positively correlated with the expressions of cell-cycle genes. Finally, we experimentally confirmed the role of therapy-resistant lncRNA SNHG1, and therapy-sensitive lncRNA UBL7-AS1 in promoting cell proliferation in human GBM U138MG cells.

## 2. Materials and methods

### 2.1. Microarray data acquisition

Microarray datasets (GSE50161, GSE4290, and GSE7696) were obtained from the Gene Expression Omnibus (GEO) database of NCBI (http://www.ncbi.nlm.nih.gov/geo/) [16, 17]. The statistics and description of the datasets are shown in Supplemental Table 1. All datasets used in this study were generated on the microarray platform GPL570 [HG-U133_Plus_2] Affymetrix Human Genome U133 Plus 2.0 Array. The raw data were normalized with the Robust Multichip Average (RMA) method using the R software limma package.

### 2.2. Identification of differentially expressed lncRNAs

The GEO2R (R 3.2.3, Biobase 2.30.0, GEOquery 2.40.0, limma 3.26.8) [17] web tool (http://www.ncbi.nlm.nih.gov/geo/geo2r/) was used to identify differentially expressed genes between two given groups of samples in a GEO profile. lncRNAs with p ≤0.05 and |(log fold change)| ≥1 were selected for further analysis. Expression of lncRNAs in GBM was further validated in the TCGA database [18].

### 2.3. Functional enrichment analysis of lncRNAs based on their correlated mRNAs

Pathway enrichment analysis on lncRNA-correlating genes was performed using the R2 KEGG Pathway Finder by gene correlation (R2: Genomics Analysis and Visualization Platform (http://r2.amc.nl) using the dataset GSE7696. Genes with a p-value <0.05, present calls > =1 (transform_log2) were considered as lncRNA-correlating genes. Pathways with P-value <=0.01 (cutoff 0.01) were considered significant over-representation in the dataset and were ranked by the sum of the negative log10 p-value of each lncRNA for each pathway.

### 2.4. Cell cultures

Human GBM U138MG (HTB-16™, ATCC) cells were maintained in Dulbecco's Modified Eagle Medium (DMEM) high glucose supplemented with 10% heat-inactivated fetal bovine serum (FBS), glutamine (2 mM), penicillin (100 U/mL), and streptomycin (100 *μ*g/mL). U138MG cells were used within 15 passages. Human primary astrocytes were from ScienCell Research Laboratories (Carlsbad, CA, USA), cultured in astrocyte medium (ScienCell), and used within 12 passages.

### 2.5. Small interfering RNA (siRNA) transfection

The siRNAs used in this study are listed in Table 1. Cells were transfected with 30 nM siRNA using lipofectamine RNAiMAX (Invitrogen) in serum-free Opti-MEM according to the manufacturer's instructions.

### 2.6. Real-time PCR

According to the manufacturer's instructions, cDNA was synthesized using a Verso cDNA kit (AB-1453/B; Thermo Fisher Scientific). Real-time PCR was performed using SYBR Green ROX qPCR Master Mix (QIAGEN, 330510) using the primers listed in Table 1. The comparative cycle threshold (Ct) method (2^*ΔΔ*Ct) was used to calculate the relative level of gene expression. The Ct values were normalized to GAPDH, which served as an internal control.

### 2.7. Western blotting

Cells were lysed using a mammalian cell lysis kit (Sigma-Aldrich), as described previously [19]. Proteins were separated in an SDS-polyacrylamide gel followed by transfer to a PVDF membrane. The membrane was blocked with 3% nonfat dry milk, 0.05% Tween 20 in Tris-buffered saline (TBS, 150 mM NaCl, 10 mM Tris-HCl, pH 8) (TTBS) for 1 h at room temperature (RT). The membrane was then probed with primary antibody in 5% nonfat milk overnight at 4°C. Primary antibodies specific for MAD2L1 (1 : 1,000; Proteintech), CCNB2 (1 : 1,000; Proteintech) and *β*-actin (1 : 6,000; Proteintech) were used in this study. Next day, the membrane was washed three times with TTBS for 10 min each and subsequently incubated with secondary antibody – alkaline phosphatase-conjugated to goat anti-mouse/rabbit IgG (1 : 10,000; Jackson ImmunoResearch Labs) for 1 h at RT. The membrane was washed three times with TTBS for 10 min each and then developed using West Chemiluminescent Substrate (Thermo Fisher Scientific). All experiments were repeated at least three times, and representative blots are presented in the figures.

### 2.8. Immunostaining

Cells cultured on slides or coverslips were fixed with 4% paraformaldehyde for 10 min at room temperature, followed by permeabilization with 0.3% Triton X-100 in PBS. Sections were incubated with a blocking buffer containing 5% BSA in PBS for 1 h at room temperature, followed by addition of rabbit anti-Ki67 (Proteintech) and incubated overnight at 4°C. Primary Abs were labeled with secondary Abs conjugated to the fluorescent probes, and nuclei were labeled with DAPI. Slides were covered with a coverslip with ProLong Gold antifade reagent (Invitrogen) and allowed to dry for 24 h at room temperature. Images were captured with a 20X objective.

### 2.9. Cell proliferation assays

Cells were seeded in the 96-well plate with a density of 5000 cells per well. Cell proliferation assays were performed after transfecting siRNA. Cell proliferation was assessed using the CyQUANT™ Cell Proliferation Assay Kit (Invitrogen) according to the manufacturer's instructions.

### 2.10. Statistical analysis and figure generation

P-values were calculated using either a two-tailed unpaired t-test or one-way analysis of variance (ANOVA) for differential expression as indicated in the figure legends. Boxplots and scatter plots figures were generated using GraphPad Prism version 6.01 for Windows (GraphPad Software). Venn diagrams were generated using the Venny tool at http://bioinformatics.psb.ugent.be/webtools/Venn/. Heatmaps were generated using Morpheus, https://software.broadinstitute.org/morpheus.

## 3. Results

### 3.1. Identification of dysregulated lncRNAs in various human brain tumors compared with normal brain tissues

**Figure 1:**
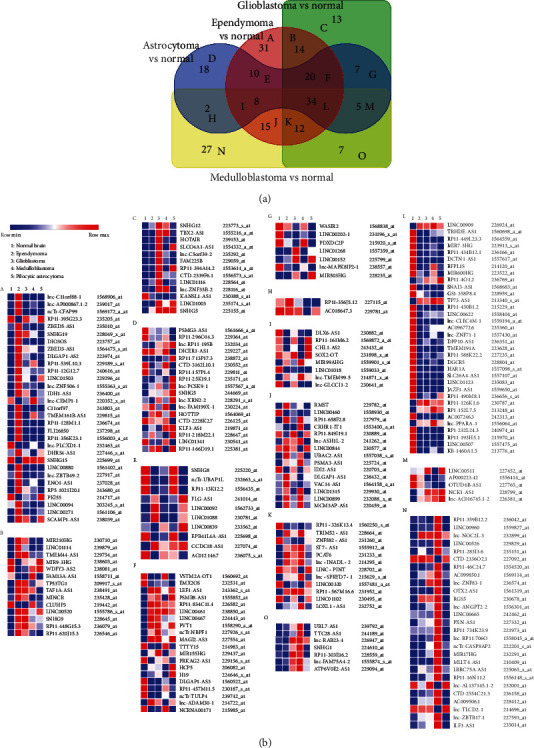
Dysregulated lncRNAs in various brain tumors compared with normal brain tissues. (a) Venn diagram showing dysregulated lncRNAs by1.0-fold or greater (p <0.05) in brain tumors such as ependymoma (n =46), glioblastoma (n =34), medulloblastoma (n =22), and pilocytic astrocytoma (n =15) compared with control brain tissues (n =13) from dataset GSE50161. (b) (A-O) Heatmap illustrating the expression (average log expression value) of the dysregulated lncRNAs between indicated brain tumor and normal brain samples in a. (a and b-A, C, D, N) The expressions of 31, 13, 18, and 27 lncRNAs were exclusively dysregulated in ependymoma, GBM, PA, and medulloblastomas tumors, compared with control samples, respectively. (a and b-B, E, G, H, J, O) The expressions of 14, 10, 7, 2, 15, and 7 lncRNAs were dysregulated in both ependymoma and GBM; ependymoma and PA; GBM and PA; PA and medulloblastomas; ependymoma and medulloblastomas; GBM and medulloblastomas; compared with control samples, respectively. (a and b-F, I, K) The expressions of 20, 8, and 12 lncRNAs were dysregulated in ependymoma, GBM and PA; ependymoma, PA and medulloblastomas; GBM, ependymoma and medulloblastomas, compared with control samples, respectively.

To identify dysregulated lncRNAs in human brain tumors, we first examined the dataset GSE50161, which contains brain samples from 46 ependymomas, 34 GBM, 15 pilocytic astrocytoma (PA), and 22 medulloblastomas,, and 13 normal brain samples [20]. The expressions of 31, 13, 18, and 27 lncRNAs were exclusively dysregulated in ependymoma, GBM, PA, and medulloblastomas tumors, compared with control samples, respectively (Figures 1(a) and 1(b)-a, c, d, n). The expressions of 14, 10, 7, 2, 15, and 7 lncRNAs were dysregulated in both ependymoma and GBM; ependymoma and PA; GBM and PA; PA and medulloblastomas; ependymoma and medulloblastomas; GBM and medulloblastomas; compared with control samples, respectively (Figures 1(a) and 1(b)-b, e, g, h, j, o). The expressions of 20, 8, and 12 lncRNAs were dysregulated in ependymoma, GBM and PA; ependymoma, PA and medulloblastomas; GBM, ependymoma and medulloblastomas, compared with control samples, respectively (Figures 1(a) and 1(b)-f, i, k). Moreover, we identified 34 lncRNAs commonly aberrantly expressed in all tumor samples compared with control samples simultaneously (Figures 1(a) and 1(b)-l).

### 3.2. Identification of dysregulated lncRNAs in various human brain tumors compared with non-tumor brain tissues from epilepsy patients

**Figure 2:**
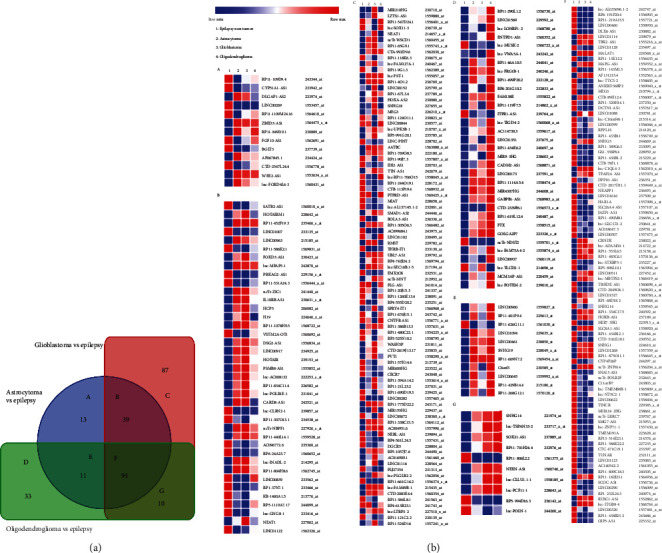
Dysregulated lncRNAs in various brain tumors compared with epilepsy non-tumor control brain tissues. (a) Venn diagram showing dysregulated lncRNAs by 1.0-fold or greater (p <0.05) in brain tumors such as astrocytoma (n =46), glioblastoma (n =81), and oligodendroglioma (n =50) compared with control brain tissues from epilepsy patients (n =23) from dataset GSE4290. (b (A-G) Heatmap illustrating the expression (average log expression value) of the dysregulated lncRNAs between indicated brain tumor and epilepsy non-tumor brain samples in a. (a and b-A, C, D) There were 13, 87, and 33 lncRNAs, respectively, differentially expressed in astrocytomas, GBM, and oligodendrogliomas compared with nontumor epilepsy brain samples. (a and b-B, E, G) There were 39, 11, and 10 lncRNAs, differentially expressed in astrocytomas and GBM; astrocytomas and oligodendrogliomas; GBM and oligodendrogliomas; respectively, compared with non-tumor epilepsy brain samples. (a and b-F) The expressions of 94 lncRNAs were commonly dysregulated in all tumor samples compared with non-tumor epilepsy brain samples simultaneously.

We next sought to examine the expression of lncRNAs in another dataset – GSE4290 – which contains 23 non-tumor control samples from epilepsy patients, 26 astrocytomas (grade 2-3), 81 GBM [21], and 50 oligodendrogliomas. There were 13, 87, and 33 lncRNAs, respectively, differentially expressed in astrocytomas, GBM, and oligodendrogliomas compared with nontumor epilepsy brain samples (Figures 2(a) and 2(b)-a, c, d). There were 39, 11, and 10 lncRNAs, differentially expressed in astrocytomas and GBM; astrocytomas and oligodendrogliomas; GBM and oligodendrogliomas; , respectively, compared with non-tumor epilepsy brain samples (Figures 2(a) and 2(b)-b, e, g). The expressions of 94 lncRNAs were commonly dysregulated in all tumor samples compared with non-tumor epilepsy brain samples simultaneously (Figures 2(a) and 2(b)-f).

### 3.3. Dysregulated lncRNAs in GBM

**Figure 3:**
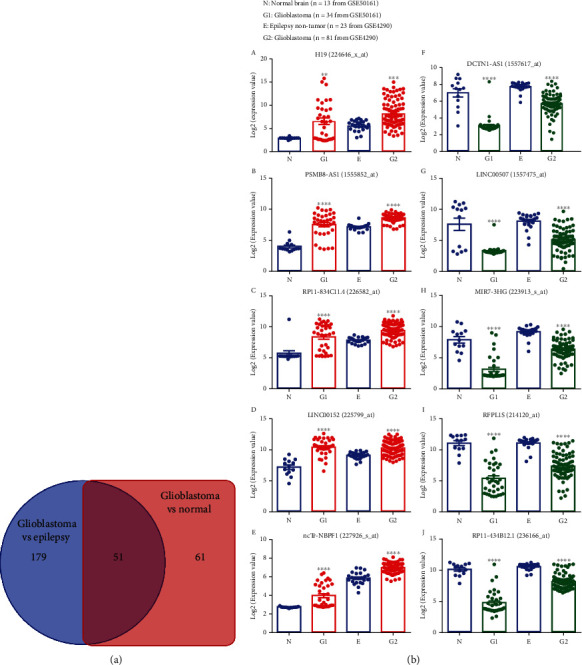
Dysregulated lncRNAs in glioblastoma. (a) Venn diagram showing commonly dysregulated lncRNAs by 1.0-fold or greater (p <0.05) in both datasets GSE50161 and GSE4290. (b) (A-J) Boxplots of expression levels of selected up- and down-regulated lncRNAs in glioblastoma compared with normal or epilepsy non-tumor brain tissues. P-values were calculated using on-way ANOVA where: ∗∗ p <0.01, ∗∗∗ p <0.001, ∗∗∗∗ p <0.0001, G1 vs N and G2 vs E.

Having determined the dysregulated lncRNAs in GBM in two unrelated studies, we next sought to find dysregulated lncRNAs in both datasets. As shown in Figure 3(a), there were 51 lncRNAs that aberrantly expressed in GBM compared with either normal control or non-tumor epilepsy controls. Interestingly, among them, 30 and 21 lncRNAs were, respectively, up- and down-regulated in GBM in both datasets (Supplemental Table 2). The representative up- and down-regulated lncRNAs in GBM are shown in Figures 3(a) and 3(b)a-j. We further validated these findings in TCGA databases [18]. The expression of SNHG1and UBL7-AS1 were up-regulated, while the VSTM2A-OT1 and EMX2OS were down-regulated in GBM cases from public TCGA databases (Supplemental Figures 1).

### 3.4. Identification of therapy-resistant and therapy-reversed lncRNAs in GBM

**Figure 4:**
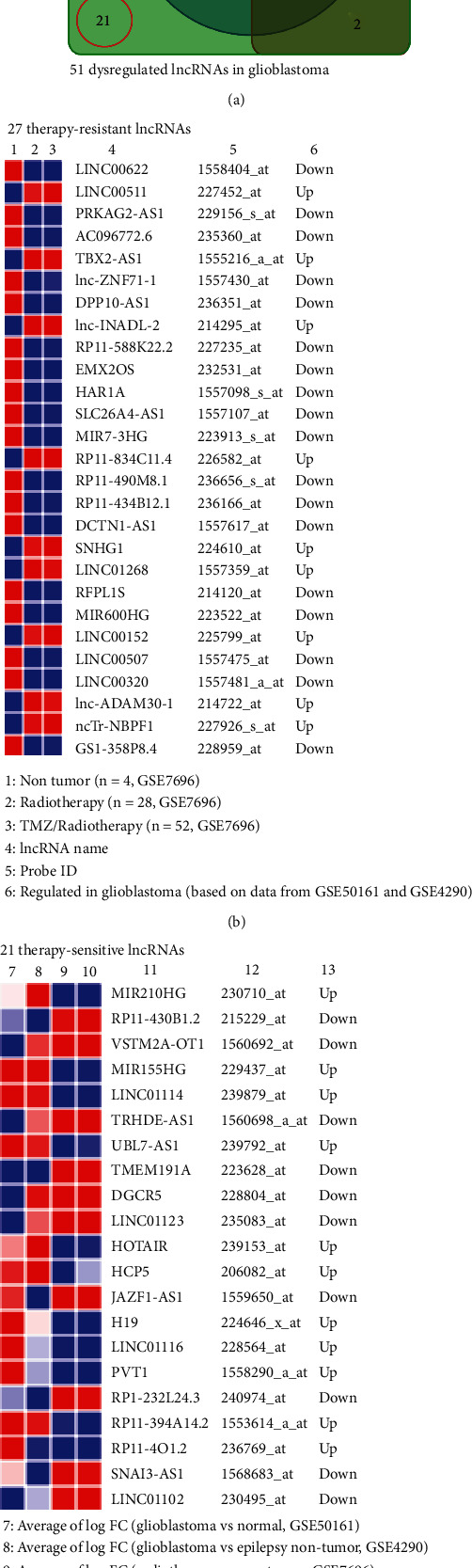
Identification of therapy-resistance and sensitive lncRNAs in GBM. (a) Venn diagram illustrating radiotherapy and TMZ/radiotherapy (21) and therapy-resistant lncRNAs (27) according to dataset GSE7696. One lncRNA LINC-PINT remained down-regulated in GBM of patients who received radiotherapy, but its expression was reversed in GBM of patients who received TMZ/radiotherapy compared with non-tumor brain tissues. The expression of two lncRNAs – PSMB8-AS1 and KB-1460A1.5 – was reversed in GBM of patients who received radiotherapy but remained down-regulated in GBM of patients who received TMZ/radiotherapy compared with non-tumor brain tissues. (b) Heatmap showing the expression (average log expression value) of therapy-resistant dysregulated lncRNAs in glioblastoma. (c) Heatmap showing the expression (average log expression value) of therapy-sensitive dysregulated lncRNAs in glioblastoma. (a and b) The expressions of 27 GBM-associated lncRNAs (identified as above) remained either up- or down-regulated in GBM of patients who received with either radiotherapy and TMZ/radiotherapy compared with control samples. (a and c) Both radiotherapy and TMZ/radiotherapy successfully reversed the expression of 21 lncRNAs in GBM.

Owing to the poor outcome of the therapies for GBM, we proposed that GBM-associated lncRNAs that were resistant to therapies could be related to treatment noncompliance. For this, we examined the expression of the 51 GBM-associated lncRNAs in another dataset GSE7696. This dataset contains 4 non-tumor brain tissue samples, 28 GBM specimens from patients treated with radiotherapy, and 52 GBM specimens of patients treated with adjuvant temozolomide (TMZ) and radiotherapy [22, 23]. Intriguingly, the expressions of 27 GBM-associated lncRNAs (identified as above) remained either up- or down-regulated in GBM of patients who received with either radiotherapy and TMZ/radiotherapy compared with control samples (Figures 4(a) and 4(b)), while both radiotherapy and TMZ/radiotherapy successfully reversed the expression of 21 lncRNAs in GBM (Figures 4(a) and 4(c)). Additionally, one lncRNA LINC-PINT remained down-regulated in GBM of patients who received radiotherapy, but its expression was reversed in GBM of patients who received TMZ/radiotherapy compared with non-tumor brain tissues (Figures 4(a)). The expression of two lncRNAs, PSMB8-AS1 and KB-1460A1.5 was reversed in GBM of patients who received radiotherapy but remained down-regulated in GBM of patients who received TMZ/radiotherapy compared with non-tumor brain tissues (Figures 4(a)).

### 3.5. Functional analysis of therapy-resistant and -reversed lncRNAs

To investigate the potential functions of the therapy-resistant and -reversed lncRNAs in GBM, pathway enrichment analysis on lncRNA-correlating genes was performed using the R2 KEGG Pathway Finder. Data used for the correlation analyses were from the GSE7696 dataset in the R2 platform. The functional pathways were ranked by the sum of each lncRNA's negative log10 p-value for each pathway. The top 30 pathways correlated with therapy-resistant and -reversed lncRNAs are shown in Supplemental Figures 2A and 2B. Interestingly, the expressions of therapy-resistant and -reversed lncRNAs such as SNHG1, GS1-358P8.4 (Supplemental Figure 2A), UBL7-AS1, and RP11-4O1.2 were significantly correlated with the expression of cell-cycle genes in the dataset, suggesting both therapy-resistant and -reversed lncRNAs could play a role in GBM proliferation.

**Figure 5:**
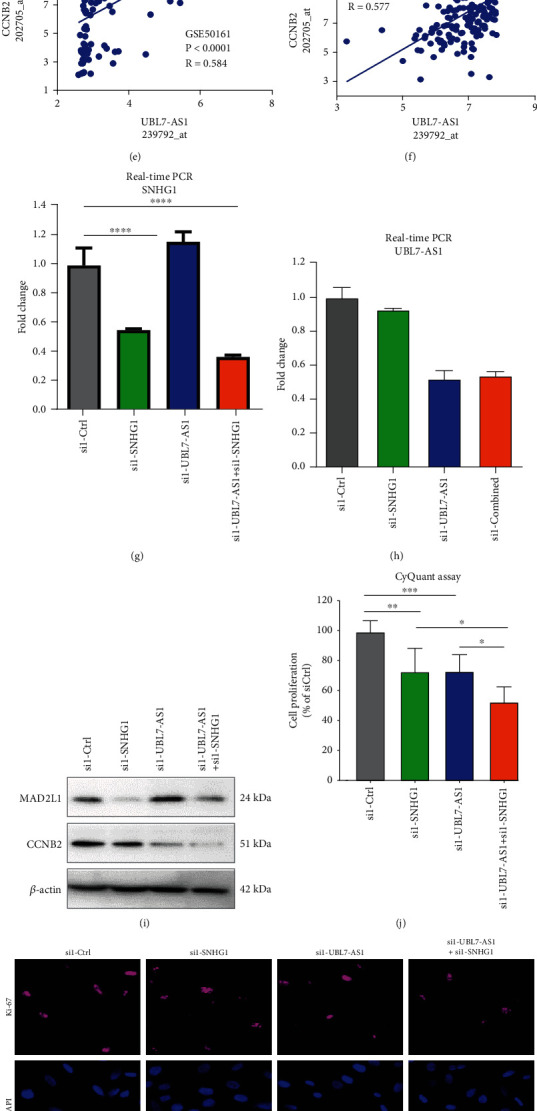
Knockdown of SNHG1 and UBL7-AS1 decreases proliferation of U138MG cells. Expression levels of glioblastoma associated lncRNAs correlate with the expression of cell cycle genes. (a) Vulcano correlation plot showing the expression cell-cycle genes positively and negatively correlated with SNHG1 in dataset GSE7696. (b and c) Scatter plots for log expression of lncRNA expression (*x*-axis) versus log expression of the most positively correlated cell-cycle gene – MAD2L1 in dataset GSE50161 (d) and GSE4290 (e). (d) Vulcano correlation plot showing the expression cell-cycle genes positively and negatively correlated with UBL7-AS1 in dataset GSE7696. (e and f) Scatter plots for log expression of lncRNA expression (x-axis) versus log expression of the most positively correlated cell-cycle gene – CCNB2 in dataset GSE50161 (g) and GSE4290 (h). (g and h) Real-time PCR analysis of the expression of SNHG1 (a) and UBL7-AS1 (b) in U138MG cells transfected with siRNA to SNHG1, UBL7-AS1, or both. (i) Western blotting analysis of the expression of MAD2L1 and CCNB2 in U138MG cells transfected with siRNAs to SNHG1, UBL7-AS1 or both. (j) Cell proliferation analysis in U138MG cells transfected with siRNAs to SNHG1, UBL7-AS1, or both using the CyQUANT assay. (k) Immunostaining for Ki67 in U138MG cells transfected with siRNAs to SNHG1, UBL7-AS1, or both. (l) Quantification of the results in (e). P-values were calculated using one-way ANOVA where: ∗p <0.05, ∗∗ p <0.01, ∗∗∗ p <0.001, ∗∗∗∗ p <0.0001.

Notably, the morphine addiction pathway was also ranked in the top 30 enriched pathways among therapy-resistant and -reversed lncRNA-correlating genes (Supplemental Figures 2A and 2B). This result suggests that the use of morphine could dysregulate the expression of lncRNAs, and in turn, affect morphine tolerance and addiction cellular signaling pathways [24, 25]. As shown in Figure 5(a), the expressions of most cell-cycle genes were shown to be positively correlated with the expression of SNHG1, with the strongest correlation between SNHG1 and MAD2L1 - a positive regulator of cell proliferation [26, 27]. The positive correlation between SNHG1 and MAD2L1 was further validated in datasets GSE50161 and GSE4290 (Figures 5(b) and 5(c)). Similarly, we found that the expression of one of the therapy-reversed lncRNAs UBL7-AS1 was positively correlated with most of the cell-cycle genes with the strongest correlation between UBL7-AS1 and CCNB2, another positive regulator of cell proliferation (Figures 5(d)–5(f)). These results thus indicate that therapy-resistant and -reversed lncRNAs could regulate GBM cell proliferation.

### 3.6. Both therapy-resistant and -reversed lncRNAs regulate human GBM cell proliferation

We next sought to examine the functions of SNHG1 and UBL7-AS1 using the gene silencing approach using two siRNAs against each lncRNA (Table 1). For this, human GBM U138MG cells were transfected with SNHG1, UBL7-AS1, or both siRNAs, followed by evaluation of cell proliferation by either CyQuant assay or Ki67 immunostaining. The knockdown efficiency was evaluated by real-time PCR. As shown in Figures 5(g) and 5(h), the expression of lncRNAs, SNHG1 and UBL7-AS1 was significantly down-regulated in cells transfected with corresponding siRNAs compared with cells transfected with scrambled siRNA. Additionally, knockdown of SNHG1 and UBL7-AS1 decreased the expressions of MAD2L1 and CCNB2, respectively, in U138MG cells (Figure 5(h)). As expected, knockdown of SNHG1 and UBL7-AS1 together reduced the expression of MAD2L1 and CCNB2 simultaneously in U138MG cells (Figure 5(h)). Moreover, knockdown of either SNHG1 or UBL7-AS1 significantly decreased cell proliferation in U138MG cells, evidenced by both CyQuant assay and Ki67 immunostaining assays (Figures 5(j)–5(l)). Intriguingly, U138MG cells transfected with SNHG1 and UBL7-AS1 siRNAs exhibited decelerated cell proliferation compared with single siRNA transfected cells (Figures 5(j)–5(l)). These findings were further validated using another set of siRNAs against SNHG1 and UBL7-AS1 (Supplemental Figures 3).

We next sought to examine the role of these two lncRNAs on the expression of cell cycle genes. Interestingly, knockdown of SNHG1, UBL7-AS1 or both decreased the expression of cell-cycle positive regulators including ABL1, CCNA2, CDK6, GADD45A and WEE1 but increased the expression of cell-cycle negative regulator CDKN2D in U138MG cells (Supplemental Figures 4). Furthermore, and as expected, knockdown of SNHG1 and UBL7-AS1 did not show significant effects on proliferation in human primary astrocytes (Supplemental Figures 5). These findings thus suggest that both therapy-resistant and -sensitive lncRNAs control cell proliferation in GBM, which could, in turn, contribute to the pathogenesis and development of GBM.

## 4. Discussion

In the current study, we found that 51 lncRNAs were dysregulated in human GBM tissues. Among them, 27 lncRNAs were shown to be resistant to both radiotherapy and TMZ/radiotherapy, while 21 lncRNAs were sensitive to these therapies. Functional analyses suggest that both therapy-resistant and -sensitive lncRNAs appear to be associated with the cell-cycle pathway. We also found that the expressions of therapy-resistant lncRNA SNHG1 and therapy-sensitive lncRNA UBL7-AS1 were positively correlated with the expressions of cell-cycle genes MAD2L1 and CCNB2, respectively. Using the gene silencing approach, we demonstrated that knockdown of SNHG1 and UBL7-AS1 decreased the expression of MAD2L1 and CCNB2, respectively. Moreover, knockdown of either SNHG1 or UBL7-AS1 reduced the proliferation of human GBM U138MG cells. Additionally, knockdown of SNHG1 and UBL7-AS1 showed an additive effect in reducing cell proliferation in U138MG cells. Previous studies demonstrated that SNHG1 could promote cell proliferation by acting as a sponge of miR-145, miR-143-3p, miR-194, miR-137and miR-9-5p [28–32]. Interestingly, MAD2L1 is a potential target of these miRNAs, according to Targetscan analysis [33].

Previous studies have demonstrated that numerous lncRNAs are associated with various brain disorders [24, 34, 35], including GBM, such as TP73-AS1 [36], H19 [37], HOTAIR [7], and LINC00152 [38]. In line with these studies, we found that the expression of TP73-AS1 was up-regulated in GBM compared with normal controls, while the expressions of H19, HOTAIR and LINC00152 were significantly up-regulated in GBM compared with either normal controls or epilepsy non-tumor brain samples. Previous studies have also identified a group of lncRNAs resistant to TMZ treatment in various cell lines [39]. Here, we found that 27 lncRNAs were shown to be resistant to both radiotherapy and TMZ/radiotherapy, suggesting that they could contribute to the poor outcome of patients on treatments. Our *in vitro* results further demonstrated that knockdown of therapy-resistant and -sensitive lncRNAs showed an additive reduction of cell proliferation in human GBM cells. These findings suggest that targeting both therapy-resistant and therapy-sensitive lncRNAs could improve therapeutic outcomes in GBM patients. Moreover, understanding the expression patterns of these lncRNAs in individual GBM patients could provide strategies for future personal adjunctive therapeutics for this disease.

## 5. Conclusion

We performed a comprehensive analysis of the lncRNA transcriptome in GBM and identified 27 therapy-resistant and 21 therapy-sensitive lncRNAs associated with various biological functions such as cell proliferation. We also experimentally demonstrated that both therapy-resistant and therapy-sensitive lncRNAs play a role in GBM cell proliferation. Future validation and functional studies on other therapy-resistant and therapy-sensitive lncRNAs, including levels of these lncRNAs in the plasma of GBM patients, would be valuable to extend this study.

## Figures and Tables

**(a):** 

Gene	qPCR forward primer 5'-3'	qPCR reverse primer 5'-3'
GAPDH	ACCATCTTCCAGGAGCGAGA	CACCCTGTTGCTGTAGCCAA
SNHG1	TCTGTGTTCACTCCAGGCTGA	TGCCTGAGTTTGGGTTCTGG
UBL7-AS1	ACCTCTGATTGGACTCTTCTCAAG	GCCTTCAGCTGCTACGATCA
ABL1	GGGAAATTGTCCAGGCTCAA	TCACAGCATCAACCAGACTCG
CCNA2	GGACAAAGCTGGCCTGAATCA	TGACTGTTGTGCATGCTGTGG
CDK6	TTCACACCGAGTAGTGCATCG	TGGAAACTATAGATGCGGGCA
CDKN2D	ACATGCTGCTGGAGGAGGTT	CGGTGCTGCCAAACATCAT
GADD45A	TGGCTCTGCAGATCCACTTCA	ATTCGTCACCAGCACGCAGT
WEE1	GGCTGGATGGATGCATTTATG	GCCCACGCAGAGAAATATCG

**(b):** 

siRNA target	Sense oligo (5'-3')	Antisense oligo (5'-3')
Negative control	UAAGGCUAUGAAGAGAUACUU	GUAUCUCUUCAUAGCCUUAUU
SNHG1 siRNA1	CAUGUAGGUAGCUCAUUCAUU	UGAAUGAGCUACCUACAUGUU
SNHG1 siRNA2	CAUAGCUUUAAGAGAUCCUUU	AGGAUCUCUUAAAGCUAUGUU
UBL7-AS1 siRNA1	GUUGAUCGUAGCAGCUGAAUU	UUCAGCUGCUACGAUCAACUU
UBL7-AS1 siRNA2	CCUGUAUUCUUCGGACCAUUU	AUGGUCCGAAGAAUACAGGUU

## Data Availability

The original data (GSE50161, GSE4290, and GSE7696) published by others are available at the Gene Expression Omnibus (GEO) database of NCBI (http://www.ncbi.nlm.nih.gov/geo/). Anonymized data are available from the corresponding author upon reasonable request.

## Abbreviations

LncRNA: Long non-coding RNA
GBM: Glioblastoma
TMZ: Temozolomide
SNHG1: Small nucleolar RNA host gene 1
UBL7-AS1: UBL7 antisense RNA 1
Real-time PCR: Real-time quantitative polymerase chain reaction
siRNA: Small interfering RNA.

## Supplementary Materials

Supplementary 1 Supplementary Figure 1: Expression of SNHG1, UBL7-AS1, VSTM2A-OT1 and EMX2OS in GBM patient samples as determined by analysis of the UALCAN portal.

Supplementary 2 Supplementary Figure 2: Expression levels of glioblastoma-associated lncRNAs correlate with the expressions of cell-cycle genes.

Supplementary 3 Supplementary Figure 3: Knockdown of SNHG1 and UBL7-AS1 decreases proliferation of U138MG cells.

Supplementary 4 Supplementary Figure 4: lnRNAs SNHG1 and UBL7-AS1 cell cycle-related genes in U138MG cells.

Supplementary 5 Supplementary Figure 5: Knockdown of SNHG1 and UBL7-AS1 does not affect proliferation of human primary astrocytes.

Supplementary 6 Supplementary Table 1: The statistics and description of the datasets used in this study.

Supplementary 7 Supplementary Table 2: Fifty-one commonly dysregulated lncRNAs in glioblastoma in both datasets GSE50161 and GSE4290.
