# Supplementary material for: Therapy-resistant and -sensitive lncRNAs, SNHG1 and UBL7-AS1 promote glioblastoma cell proliferation
Source: Oxid Med Cell Longev. 2022 Mar 11;2022:2623599. doi: 10.1155/2022/2623599 (PMC8933655; doi:10.1155/2022/2623599)
Supplement: Supplementary 1 — Supplementary Figure 1: Expression of SNHG1, UBL7-AS1, VSTM2A-OT1 and EMX2OS in GBM patient samples as determined by analysis of the UALCAN portal. [file 2623599.f1.pdf]

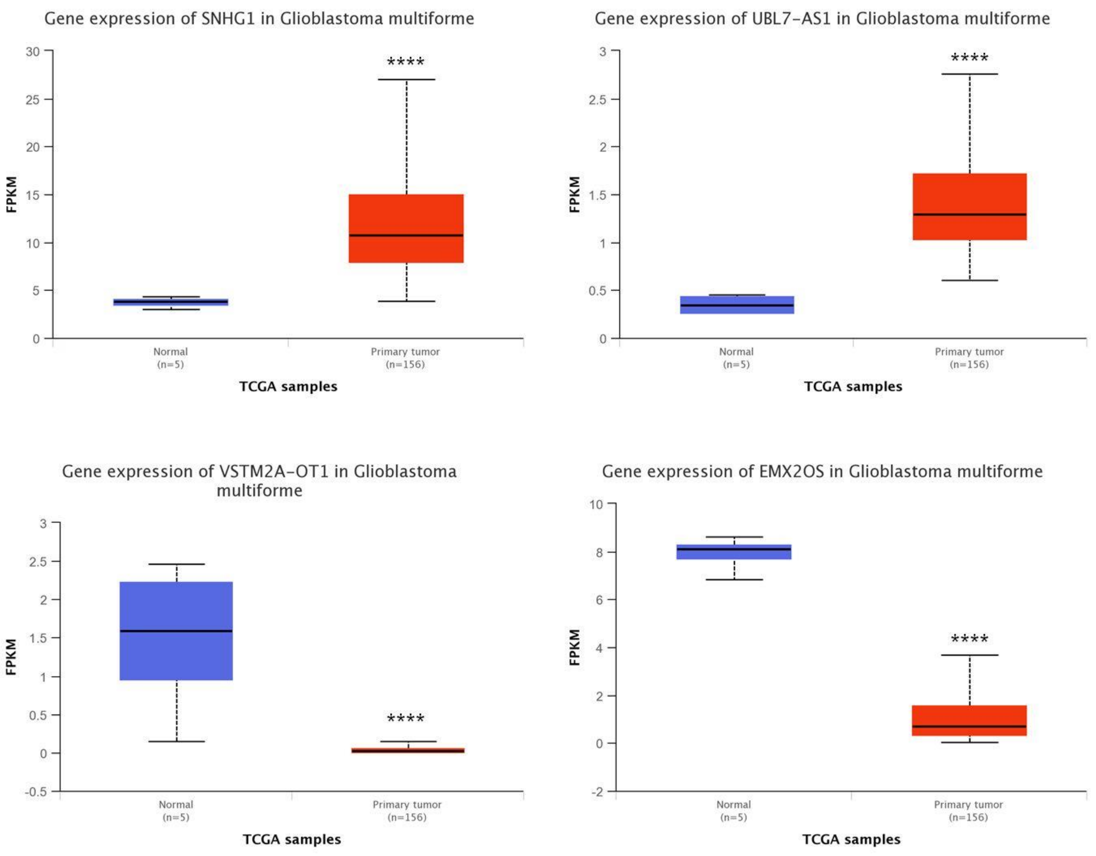


**Supplemental FIGURE 1. Expression of SNHG1, UBL7-AS1, VSTM2A-OT1 and EMX2OS in GBM patient samples as determined by analysis of the UALCAN portal.**

**(A)** Gene expression of SNHG1 in Glioblastoma multiforme. **(B)** Gene expression of UBL7-AS1 in Glioblastoma multiforme. **(C)** Gene expression of VSTM2A-OT1 in Glioblastoma multiforme. **(D)** Gene expression of EMX2OS in Glioblastoma multiforme. P-values were calculated using one-way ANOVA where:**** $p < 0.0001$.
