# Supplementary material for: Therapy-resistant and -sensitive lncRNAs, SNHG1 and UBL7-AS1 promote glioblastoma cell proliferation
Source: Oxid Med Cell Longev. 2022 Mar 11;2022:2623599. doi: 10.1155/2022/2623599 (PMC8933655; doi:10.1155/2022/2623599)
Supplement: Supplementary 2 — Supplementary Figure 2: Expression levels of glioblastoma-associated lncRNAs correlate with the expressions of cell-cycle genes. [file 2623599.f2.pdf]

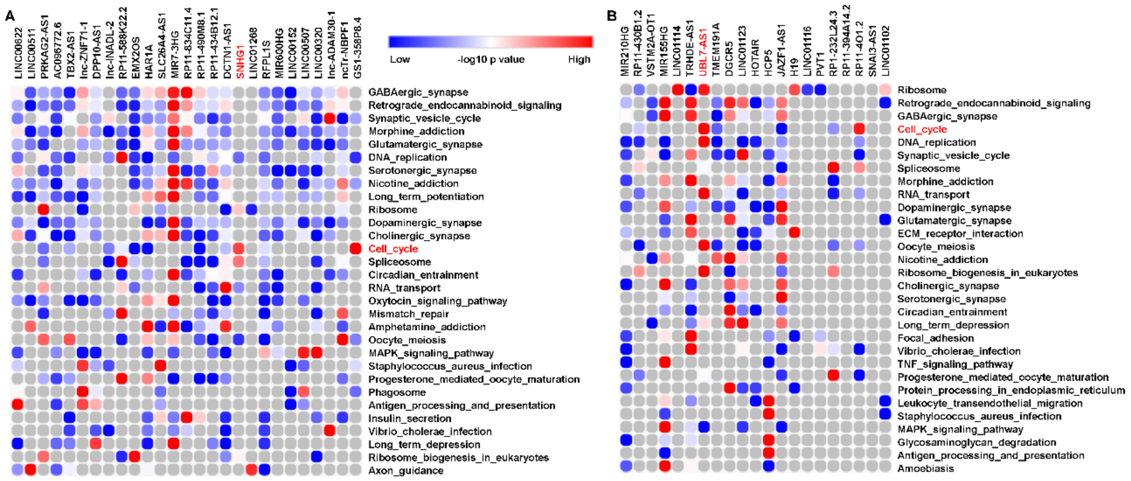

Supplemental FIGURE 2. Expression levels of glioblastoma-associated lncRNAs correlate with the expressions of cell-cycle genes.

(A) Top 30 KEGG pathways significantly correlated with the expression of therapy-resistant lncRNAs. **(B)** Top 30 KEGG pathways significantly correlated with the expression of therapy reversed lncRNAs. Each column corresponds to a single lncRNA, and each row corresponds to a KEGG pathway with an overrepresentation of genes correlating with lncRNA ($p <= 0.01$). The pathways were ranked by the sum of the negative log10 p-value of each lncRNA for each pathway. Top 30 pathways are shown.
