# Supplementary material for: Therapy-resistant and -sensitive lncRNAs, SNHG1 and UBL7-AS1 promote glioblastoma cell proliferation
Source: Oxid Med Cell Longev. 2022 Mar 11;2022:2623599. doi: 10.1155/2022/2623599 (PMC8933655; doi:10.1155/2022/2623599)
Supplement: Supplementary 3 — Supplementary Figure 3: Knockdown of SNHG1 and UBL7-AS1 decreases proliferation of U138MG cells. [file 2623599.f3.pdf]

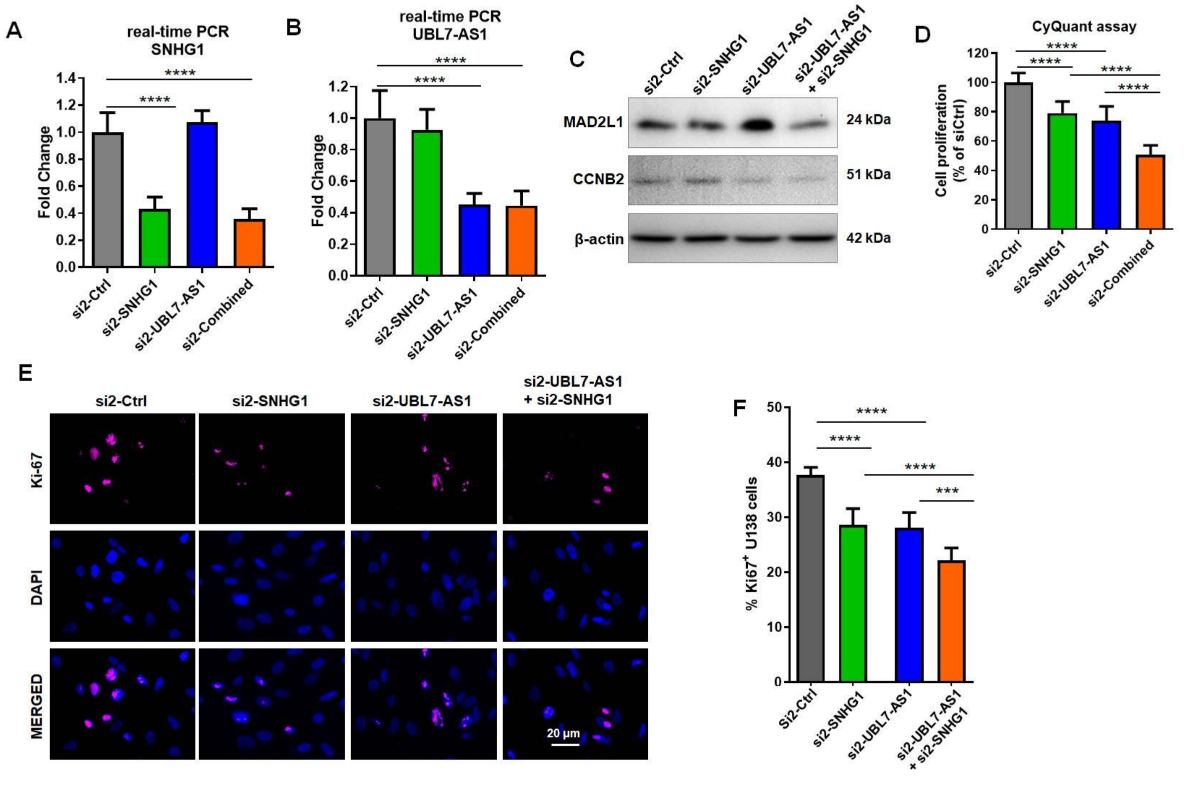

Supplemental FIGURE 3. Knockdown of SNHG1 and UBL7-AS1 decreases proliferation of U138MG cells.

(A and B) Real-time PCR analysis of the expression of SNHG1 (A) and UBL7-AS1 (B) in U138MG cells transfected with siRNA to SNHG1, UBL7-AS1 or both. **(C)** Western blotting analysis of the expression of MAD2L1 and CCNB2 in U138MG cells transfected with siRNAs to SNHG1, UBL7-AS1 or both. **(D)** Cell proliferation analysis in U138MG cells transfected with siRNAs to SNHG1, UBL7-AS1 or both using the CyQUANT assay. **(E)** Immunostaining for Ki67 in U138MG cells transfected with siRNAs to SNHG1, UBL7-AS1 or both. **(F)** Quantification of the results in E. P-values were calculated using on-way ANOVA where: *** $p < 0.001$, **** $p < 0.0001$.
