# Supplementary material for: Therapy-resistant and -sensitive lncRNAs, SNHG1 and UBL7-AS1 promote glioblastoma cell proliferation
Source: Oxid Med Cell Longev. 2022 Mar 11;2022:2623599. doi: 10.1155/2022/2623599 (PMC8933655; doi:10.1155/2022/2623599)
Supplement: Supplementary 4 — Supplementary Figure 4: lnRNAs SNHG1 and UBL7-AS1 cell cycle-related genes in U138MG cells. [file 2623599.f4.pdf]

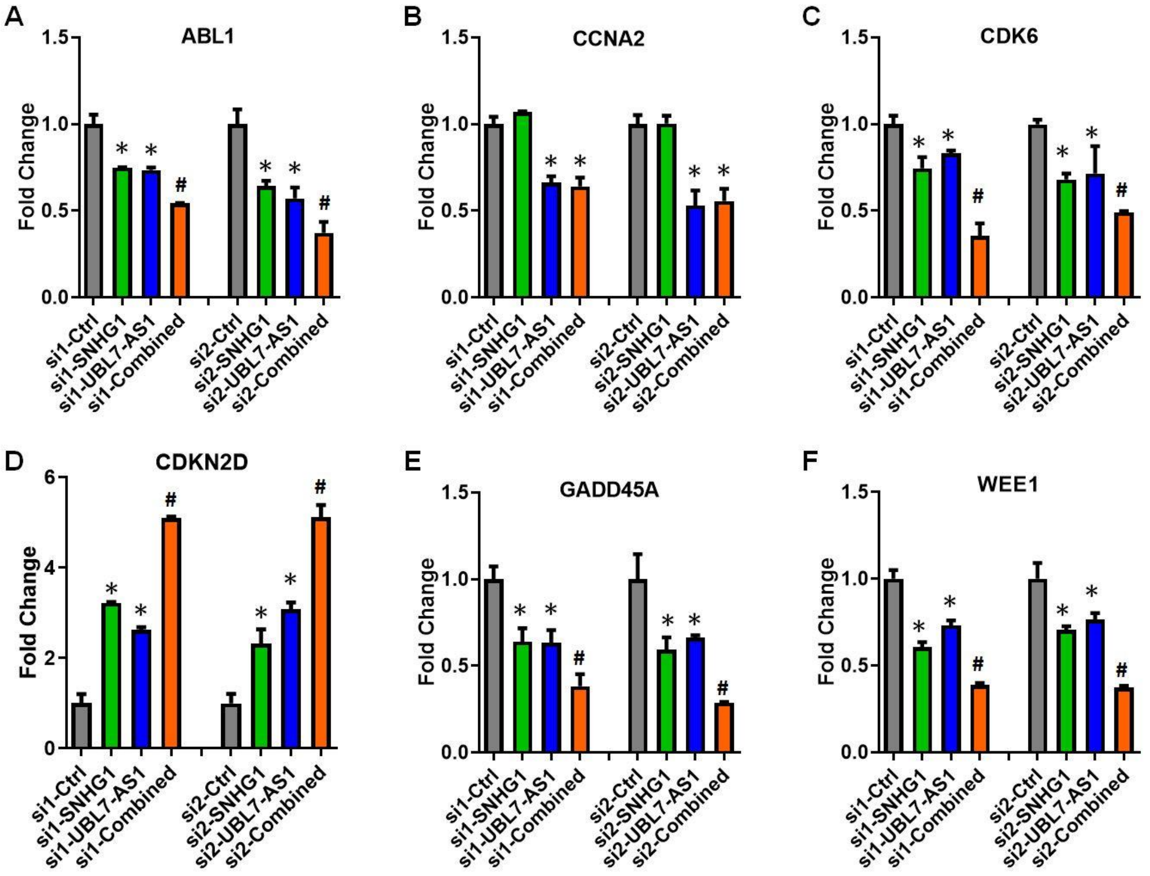

Supplemental FIGURE 4. lnRNAs SNHG1 and UBL7-AS1 cell cycle-related genes in U138MG cells.

(A - F) Real-time PCR analysis of the expression of cell cycle-related genes in U138MG cells transfected with siRNA to SNHG1, UBL7-AS1 or both. P-values were calculated using on-way ANOVA where: * $p < 0.05$ vs control, # $p < 0.05$ vs siRNA-SNHG1 and siRNA-UBL7-AS1.
