# Supplementary material for: Therapy-resistant and -sensitive lncRNAs, SNHG1 and UBL7-AS1 promote glioblastoma cell proliferation
Source: Oxid Med Cell Longev. 2022 Mar 11;2022:2623599. doi: 10.1155/2022/2623599 (PMC8933655; doi:10.1155/2022/2623599)
Supplement: Supplementary 5 — Supplementary Figure 5: Knockdown of SNHG1 and UBL7-AS1 does not affect proliferation of human primary astrocytes. [file 2623599.f5.pdf]

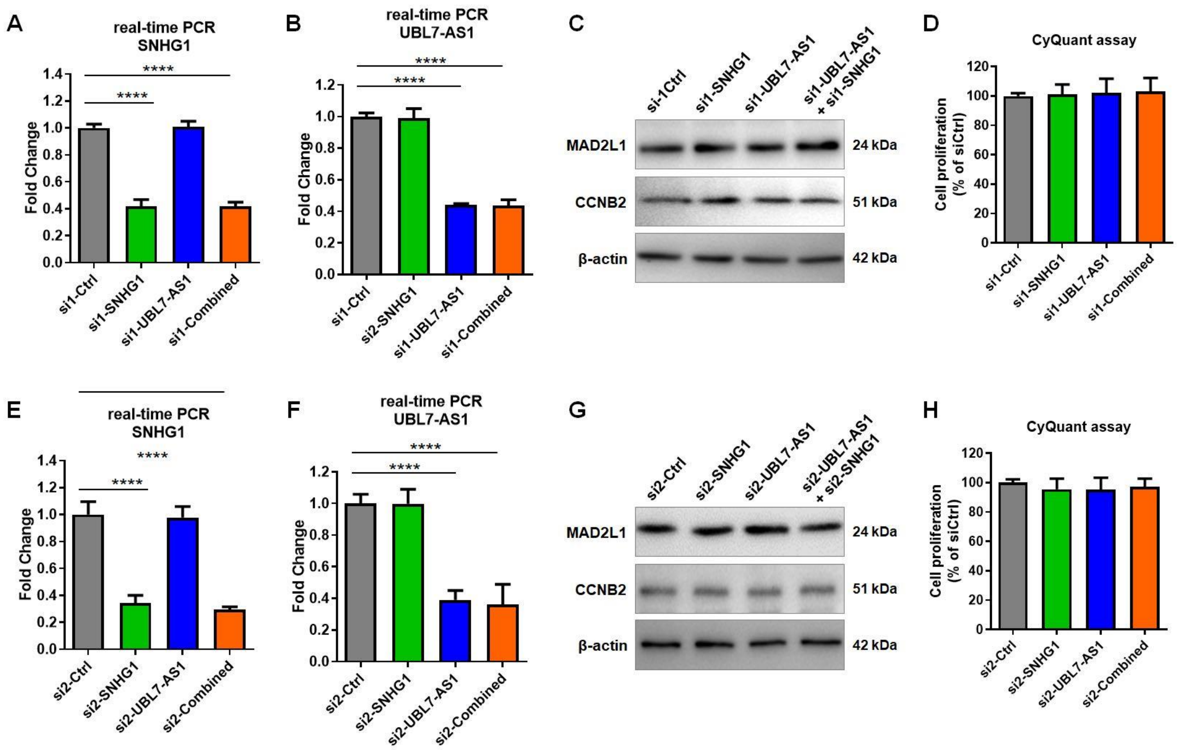

Supplemental FIGURE 5. Knockdown of SNHG1 and UBL7-AS1 doesn't affect proliferation of human primary astrocytes.

(A, B, E, F) Real-time PCR analysis of the expression of SNHG1 (A) and UBL7-AS1 (B) in human primary astrocytes transfected with siRNA to SNHG1, UBL7-AS1 or both. **(C, G)** Western blotting analysis of the expression of MAD2L1 and CCNB2 in human primary astrocytes transfected with siRNAs to SNHG1, UBL7-AS1 or both. **(D, H)** Cell proliferation analysis in human primary astrocytes transfected with siRNAs to SNHG1, UBL7-AS1 or both using the CyQUANT assay. P-values were calculated using on-way ANOVA where: *** $p < 0.001$, **** $p < 0.0001$.
