# Supplementary material for: Therapy-resistant and -sensitive lncRNAs, SNHG1 and UBL7-AS1 promote glioblastoma cell proliferation
Source: Oxid Med Cell Longev. 2022 Mar 11;2022:2623599. doi: 10.1155/2022/2623599 (PMC8933655; doi:10.1155/2022/2623599)
Supplement: Supplementary 6 — Supplementary Table 1: The statistics and description of the datasets used in this study. [file 2623599.f6.pdf]

Supplemental Table 1: The statistics and description of the datasets used in this study

GEO Datasets	Samples	References
GSE50161	13 normal brain samples, 46 ependymomas, 34 GBM, 22 medulloblastomas, and 15 pilocytic astrocytoma	[18]
GSE4290	23 non-tumor samples from epilepsy patients, 26 astrocytomas, 50 oligodendrogliomas, and 81 GBM.	[19]
GSE7696	28 GBM specimen of patients treated with radiotherapy, 52 GBM specimen of patients treated with adjuvant temozolomide (TMZ) and radiotherapy, and 4 samples of non-tumor brain tissues	[20,21]
