# Supplementary material for: Therapy-resistant and -sensitive lncRNAs, SNHG1 and UBL7-AS1 promote glioblastoma cell proliferation
Source: Oxid Med Cell Longev. 2022 Mar 11;2022:2623599. doi: 10.1155/2022/2623599 (PMC8933655; doi:10.1155/2022/2623599)
Supplement: Supplementary 7 — Supplementary Table 2: Fifty-one commonly dysregulated lncRNAs in glioblastoma in both datasets GSE50161 and GSE4290. [file 2623599.f7.pdf]

Supplemental Table 2. Fifty-one commonly dysregulated lncRNAs in glioblastoma in both datasets GSE50161 and GSE4290

	DATASET	GSE50161		GSE4290			
lncRNA name	ID	P.Value	logFC	P.Value	logFC	Gene.symbol	Gene.title
RFPL1S	214120_at	0.000	-5.7220786	0.000	-3.8370308	RFPL1S	RFPL1 antisense RNA 1
LINC00320	1557481_a_at	0.007	-1.6244718	0.000	-3.0860473	LINC00320	long intergenic non-protein coding RNA 320
LINC00507	1557475_at	0.000	-4.3201239	0.000	-3.0737302	LINC00507	long intergenic non-protein coding RNA 507
MIR7-3HG	223913_s_at	0.000	-4.8059049	0.000	-2.875604	MIR7-3HG	MIR7-3 host gene
HAR1A	1557098_s_at	0.000	-3.238709	0.000	-2.8728801	HAR1A	highly accelerated region 1A (non-protein coding)
SLC26A4-AS1	1557107_at	0.000	-3.7194729	0.000	-2.8130842	SLC26A4-AS1	SLC26A4 antisense RNA 1
TMEM191A	223628_at	0.000	-2.7283786	0.000	-2.7848504	TMEM191A	transmembrane protein 191A (pseudogene)
JAZF1-AS1	1559650_at	0.000	-1.1643842	0.000	-2.7817697	JAZF1-AS1	JAZF1 antisense RNA 1
RP11-434B12.1	236166_at	0.000	-5.4227062	0.000	-2.4895761	LOC285147	uncharacterized LOC285147
SNAI3-AS1	1568683_at	0.000	-1.2078559	0.000	-2.329242	SNAI3-AS1	SNAI3 antisense RNA 1
RP11-430B1.2	215229_at	0.000	-1.5937564	0.000	-2.1836281	LOC100129973	uncharacterized LOC100129973
DCTN1-AS1	1557617_at	0.000	-3.9458409	0.000	-2.0495064	DCTN1-AS1	DCTN1 antisense RNA 1
DPP10-AS1	236351_at	0.000	-3.0467243	0.000	-1.9959408	DPP10-AS1	DPP10 antisense RNA 1
RP11-588K22.2	227235_at	0.000	-2.6031656	0.000	-1.8572057	GUCY1A3	guanylate cyclase 1 soluble subunit alpha
RP11-490M8.1	236656_s_at	0.000	-2.4345127	0.000	-1.8512637	LOC100288911	uncharacterized LOC100288911
KB-1460A1.5	213776_at	0.000	-3.4639081	0.000	-1.8301835	LOC157562	uncharacterized LOC157562
LINC00622	1558404_at	0.000	-3.2198077	0.000	-1.7724132	LINC00622	long intergenic non-protein coding RNA 622
RP1-232L24.3	240974_at	0.000	-1.2310855	0.000	-1.7242063	LOC100506851	uncharacterized LOC100506851

lnc-ZNF71-1	1557430_at	0.000	-3.8065703	0.000	-1.6997317	SMIM17	small integral membrane protein 17
LINC01123	235083_at	0.000	-3.9396827	0.000	-1.6811895	LINC01123///LINC01106	long intergenic non-protein coding RNA 1123///long intergenic non-protein coding RNA 1106
AC096772.6	235360_at	0.000	-1.7690117	0.000	-1.5406708	PLEKHM3	pleckstrin homology domain containing M3
TRHDE-AS1	1560698_a_at	0.000	-3.600933	0.000	-1.5359174	TRHDE-AS1	TRHDE antisense RNA 1
VSTM2A-OT1	1560692_at	0.000	-3.4528555	0.000	-1.4108019	VSTM2A-OT1///VSTM2A	VSTM2A overlapping transcript 1///V-set and transmembrane domain containing 2A
PRKAG2-AS1	229156_s_at	0.005	-1.7567494	0.000	-1.1883775	PRKAG2-AS1	PRKAG2 antisense RNA 1
EMX2OS	232531_at	0.001	-2.2410409	0.000	-1.1595829	EMX2OS	EMX2 opposite strand/antisense RNA
LINC-PINT	228702_at	0.000	-1.5776107	0.000	-1.1182049	LINC-PINT	long intergenic non-protein coding RNA, p53 induced transcript
GS1-358P8.4	228959_at	0.000	-1.5865295	0.000	-1.099731	PDK3	pyruvate dehydrogenase kinase 3
LINC01102	230495_at	0.015	-1.7675883	0.000	-1.0723266	LINC01102	long intergenic non-protein coding RNA 1102
MIR600HG	223522_at	0.000	-3.2773305	0.000	-1.0720137	MIR600///MIR600HG	microRNA 600///MIR600 host gene
DGCR5	228804_at	0.000	-4.0952272	0.000	-1.0100451	DGCR5	DiGeorge syndrome critical region gene 5 (non-protein coding)
LINC01268	1557359_at	0.000	2.4945246	0.001	1.0429209	LINC01268	long intergenic non-protein coding RNA 1268
RP11-4O1.2	236769_at	0.000	2.5927374	0.000	1.0888769	LOC158402	uncharacterized LOC158402
LINC00511	227452_at	0.000	2.7758248	0.000	1.1215444	LINC00673///LINC00511	long intergenic non-protein coding RNA 673///long intergenic non-protein coding RNA 511
UBL7-AS1	239792_at	0.001	1.2399995	0.000	1.1330232	UBL7-AS1	UBL7 antisense RNA 1 (head to head)
RP11-394A14.2	1553614_a_at	0.030	1.266249	0.004	1.1555993	LINC01198	long intergenic non-protein coding RNA 1198
LINC01116	228564_at	0.008	2.7197239	0.002	1.17112	LINC01116	long intergenic non-protein coding RNA 1116
SNHG1	224610_at	0.000	1.3601162	0.000	1.1873876	SNHG1///SNORD22///SNORD25///SNORD26///SNORD27///SNORD28///SNORD30///SNORD31///SNORD29	small nucleolar RNA host gene 1///small nucleolar RNA, C/D box 22///small nucleolar RNA, C/D box 25///small nucleolar RNA, C/D box 26///small nucleolar RNA, C/D box 27///small nucleolar RNA, C/D box 28///small nucleolar RNA, C/D box 30///small nucleolar

							RNA, C/D box 31///small nucleolar RNA, C/D box 29
LINC00152	225799_at	0.000	3.2508298	0.000	1.2458158	LOC101930489///MIR4435-2HG///LINC00152	uncharacterized LOC101930489///MIR4435-2 host gene///long intergenic non-protein coding RNA 152
lnc-INADL-2	214295_at	0.005	1.8383054	0.000	1.3213208	KIAA0485	uncharacterized LOC57235
LINC01114	239879_at	0.002	1.4042408	0.000	1.3862362	LINC01114	long intergenic non-protein coding RNA 1114
PVT1	1558290_a_at	0.000	2.6282537	0.000	1.3953128	MIR1204///PVT1	microRNA 1204///Pvt1 oncogene (non-protein coding)
ncTr-NBPF1	227926_s_at	0.000	2.7324051	0.000	1.3965054	NBPF20	neuroblastoma breakpoint family member 20
HOTAIR	239153_at	0.036	1.0875604	0.001	1.4623241	HOTAIR	HOX transcript antisense RNA
PSMB8-AS1	1555852_at	0.000	3.5606113	0.000	1.4782343	PSMB8-AS1	PSMB8 antisense RNA 1 (head to head)
MIR210HG	230710_at	0.024	1.1669203	0.000	1.567502	MIR210HG	MIR210 host gene
HCP5	206082_at	0.005	1.4510557	0.000	1.599913	HCP5	HLA complex P5 (non-protein coding)
RP11-834C11.4	226582_at	0.000	2.6795611	0.000	1.6005364	LOC400043	uncharacterized LOC400043
TBX2-AS1	1555216_a_at	0.011	1.8046834	0.000	1.7596987	LOC101060604///SLC7A5P2///SLC7A5P1	putative L-type amino acid transporter 1-like protein IMAA///solute carrier family 7 member 5 pseudogene 2///solute carrier family 7 member 5 pseudogene 1
lnc-ADAM30-1	214722_at	0.000	1.4423181	0.000	2.0086839	NOTCH2NL	notch 2 N-terminal like
MIR155HG	229437_at	0.003	2.0848099	0.000	2.0263015	MIR155///MIR155HG	microRNA 155///MIR155 host gene
H19	224646_x_at	0.002	3.6363716	0.000	2.7537401	MIR675///H19	microRNA 675///H19, imprinted maternally expressed transcript (non-protein coding)
